# Homotypic and Heterotypic Adhesion Induced by Odorant Receptors and the β2-Adrenergic Receptor

**DOI:** 10.1371/journal.pone.0080100

**Published:** 2013-12-02

**Authors:** Marion Richard, Sophie Jamet, Coralie Fouquet, Caroline Dubacq, Nicole Boggetto, Frédéric Pincet, Christine Gourier, Alain Trembleau

**Affiliations:** 1 CNRS UMR 7102, Université Pierre et Marie Curie Paris 06, Team Development and Plasticity of Neural Networks, Paris, France; 2 Imaging Facility (ImagoSeine), Jacques Monod Institute, CNRS-Université Paris Diderot, Paris, France; 3 Laboratoire de Physique Statistique, Ecole Normale Supérieure, UPMC Univ Paris 06, Université Paris Diderot, CNRS, Paris, France; Duke University, United States of America

## Abstract

In the mouse olfactory system regulated expression of a large family of G Protein-Coupled Receptors (GPCRs), the Odorant Receptors (ORs), provides each sensory neuron with a single OR identity. In the wiring of the olfactory sensory neuron projections, a complex axon sorting process ensures the segregation of >1,000 subpopulations of axons of the same OR identity into homogeneously innervated glomeruli. ORs are critical determinants in axon sorting, and their presence on olfactory axons raises the intriguing possibility that they may participate in axonal wiring through direct or indirect trans-interactions mediating adhesion or repulsion between axons. In the present work, we used a biophysical assay to test the capacity of ORs to induce adhesion of cell doublets overexpressing these receptors. We also tested the β2 Adrenergic Receptor, a non-OR GPCR known to recapitulate the functions of ORs in olfactory axon sorting. We report here the first evidence for homo- and heterotypic adhesion between cells overexpressing the ORs MOR256-17 or M71, supporting the hypothesis that ORs may contribute to olfactory axon sorting by mediating differential adhesion between axons.

## Introduction

In the mouse olfactory system, odorants are detected by Olfactory Sensory Neurons (OSNs) in the olfactory epithelium. Each OSN expresses only one Odorant Receptor (OR) gene out of a repertoire of ≈1,000 functional genes [1], [2]. ORs are G protein-coupled receptors (GPCRs) concentrated in OSN dendrites where they interact with odorants and activate a cAMP signaling pathway [1], [3]. Although OSNs expressing the same OR are dispersed across large areas of the olfactory epithelium, their axons fasciculate homotypically as they progress over the surface of the olfactory bulb (OB), and they converge into a limited number of glomeruli in the OB [4], [5]. As a consequence, adult glomeruli are homogeneously innervated by homotypic axons [5]. Very interestingly, the sorting and convergence of OSN axons relies primarily on axon-axon interactions, rather than on interactions with target cells in the OB, since OSN axon sorting and convergence occur even in absence of the OB [6].

Wiring abnormalities induced by manipulations of an ORs' amino acid sequence demonstrated that ORs are critical determinants of axon sorting (reviewed in [7]). However, the mechanisms by which ORs control OSN axons sorting have been a matter of debate. The axon sorting defects induced by manipulations of the cAMP cascade [8], [9], [10], [11] and the identification of adhesion and guidance factors, whose expression is regulated by the OR signaling pathway [11], led to a model in which each subpopulation of OSNs is endowed with a specific repertoire of adhesive/repulsive molecules through a specific level of activity of its OR-dependent cAMP cascade. According to this view, this repertoire of guidance molecules would further allow all axons of a given OR identity to fasciculate and converge. However, as this model relies essentially on the cAMP cascade downstream of ORs, it implies that this pathway could create >1,000 unique axonal identities, a hypothesis that is difficult to conceive [12]. Feinstein and Mombaerts (2004) proposed an alternative model, supported by the presence of ORs at the level of OSN axons [13], [14], [15], in which direct or indirect homophilic and heterophilic interactions mediating adhesion between ORs may underlie OSN axon sorting.

To develop an effective model suitable to investigate the adhesiveness provided by ORs, we took advantage of a biophysical assay called the dual micropipette assay, which allows measuring the force necessary to separate two adhering cells. We provide here the first strong evidence for homotypic adhesion between cells overexpressing ORs (MOR256-17 and M71) or the β2-Adrenergic Receptor (β2AR, a non-OR GPCR that can substitute to an OR in axon sorting when expressed in OSNs) [8], [16]. We also report heterotypic adhesion between cells expressing two different ORs, or one OR for one cell and the β2AR for the other cell. Collectively, our data support the hypothesis that ORs contribute to olfactory axon sorting by controlling their adhesion.

## Materials and Methods

### Plasmid constructs

pCAGGS-FLAGRhoMOR256-17-iresGFP and pCAGGS-FLAGRhoM71-iresGFP were obtained by subcloning FLAGRho from pLNCX2-FLAGRhoβ2AR-iresTauGFP (provided by S. Firestein, Columbia University, NY, USA) [8] into pCAGGS-iresGFP (provided by S. Garel, ENS, Paris, France) [17], and insertion of the MOR256-17 or M71 coding sequences PCR-amplified from genomic DNA. The presence of an Internal Ribosome Entry Site (IRES) sequence enables the expression of the OR and GFP from a single mRNA. Similarly, pCAGGS-FLAGRhoα7-5HT3-iresGFP was obtained using, instead of the OR coding sequences, the α7-5HT3 coding sequence PCR-amplified from α7-5HT-pmt2001 (provided by P.-J. Corringer and U. Maskos, Pasteur Institute, Paris, France) [18]. pCAGGS-iresGFP (CTRL) was used as a control for transfection and adhesion experiments. FLAGRhoβ2AR (containing β2AR without GFP) was made from pLNCX2-FLAGRhoβ2AR-iresTauGFP by excising the TauGFP sequence. The pCI-RTP1S plasmid was provided by H. Matsunami and J. Mainland (Duke University, Durham, USA) [19].

### Cell transfection

Murine Sarcoma 180 (S180, ATCC) cells were grown in DMEM with Glutamax (Invitrogen) supplemented with 10% Fetal Bovine Serum (FBS) (Gibco) and 100 U/ml penicillin/streptomycin (Gibco). Cells were grown at 37°C in a humidified 5% CO_2_ atmosphere and were split at regular intervals with 0.05% trypsin-EDTA (Gibco), without exceeding 25 passages. Cells were transfected at 70% confluence using Lipofectamine 2000 (Invitrogen) following the manufacturer's protocol (0.8 µg plasmid/3.2 µl lipofectamine). Cotransfections were performed at equimolar concentration of plasmids. Cells were used for further experiments at 24 h post-transfection.

### Immunocytochemistry

Antibodies used were mouse anti-FLAG (Sigma F1804), mouse anti-NeuN (Chemicon MAB377) and donkey anti-mouse DyLight 549 (Jackson Laboratories).

For “fixed” staining, cells were fixed for 15 min in 4% paraformaldehyde (PFA) in phosphate buffered saline (PBS). Each step was then followed by rinses in PBS. Cells were successively incubated for 10 min in 50 mM NH_4_Cl in PBS, permeabilized for 4 min with 0.1% Triton X-100 (Sigma) diluted in PBS and incubated for 30 min with a blocking solution composed of 5% Bovine Serum Albumin (BSA, Cohn fraction V, Sigma) in PBS. Primary antibody (anti-FLAG 1∶1,000 in blocking solution) was applied for 2 h at RT, followed by secondary antibody and Dapi (1∶1,000 in blocking solution) for 45 min. “Live” staining (without permeabilization) was performed on ice as previously described [20]. Antibody concentrations were anti-FLAG 1∶1,000, secondary antibody 1∶1,000.

### Cytometry analysis

Cells were rinsed with pre-warmed 37°C PBS before dissociation with a non-enzymatic cell dissociation solution (Sigma), and kept on ice during the whole staining procedure. 2×10^6^ cells were incubated in 100 µl of primary antibody solution containing anti-FLAG or isotypic control anti-NeuN diluted in PBS-FBS 2% (0.5 µg/ml) followed by a donkey anti-mouse Dylight 549 secondary antibody (1.4 µg/ml) (30 min each). After rinses, cells were re-suspended in PBS-FBS 2% for analysis (5×10^6^ cells/ml). FACS analysis and cell purification were done on a BD INFLUX 500 cell sorter (BD BioSciences) and cell images from imaging cytometry were acquired using an ImagestreamX Cell Analyser (Amnis Corporation).

### Adhesion test: separation force measurements

**Figure 1:**
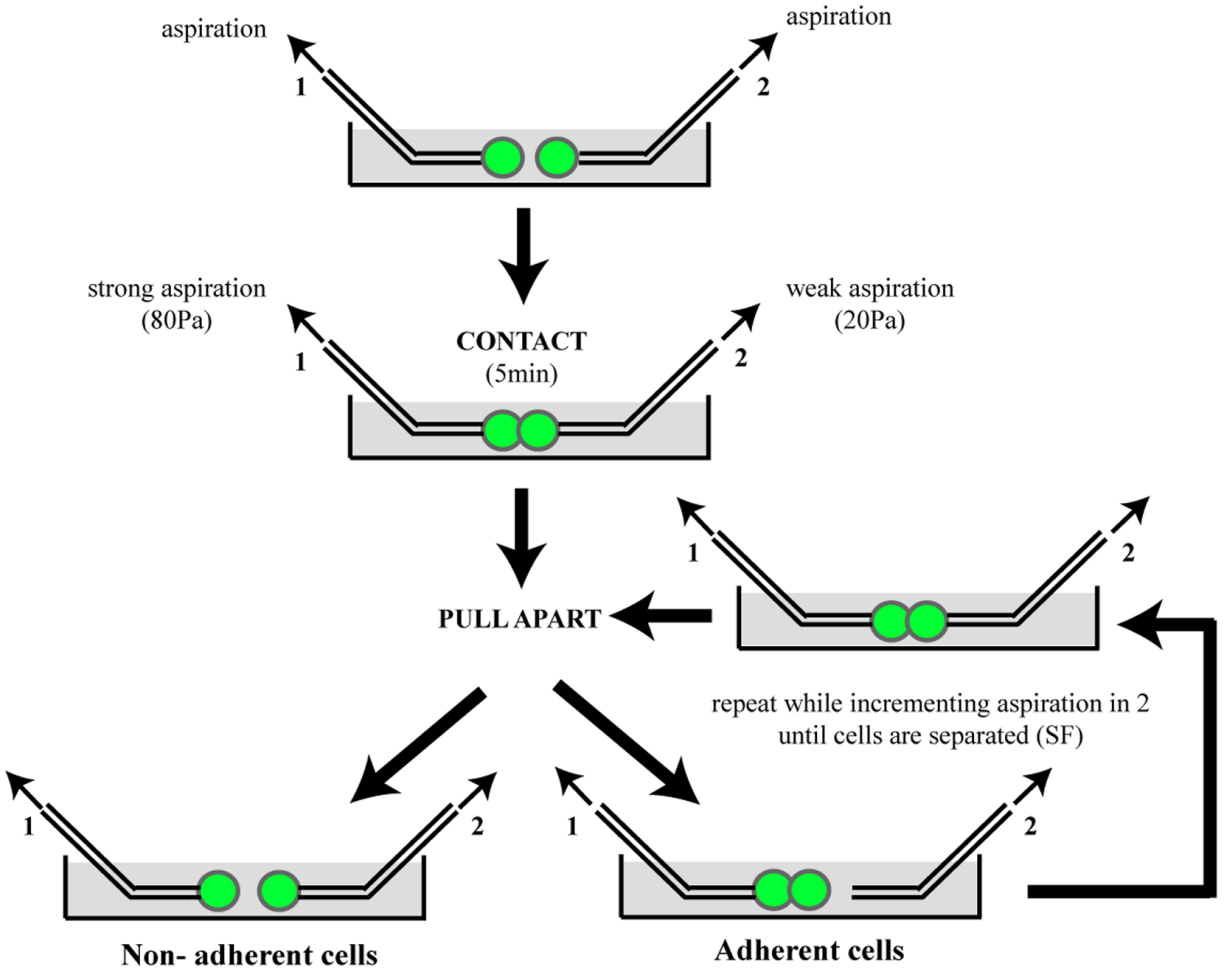
Principle of the dual pipette adhesion test. Two transfected cells held by micropipettes are challenged for adhesion by putting them in contact for several minutes and thereafter trying to pull them apart (see methods section for details). Repeating this cycle while incrementing aspiration until the cells are separated allows to measure the separation force (SF).

The experiments were performed as previously described [21]. Following dissociation (Sigma non-enzymatic solution), two GFP^+^ cells were held under controlled aspiration by micropipettes (Fig. 1). Aspiration in pipette 1 was maintained at a level high enough to hold cell 1 tightly during the whole experiment. Aspiration level in pipette 2 was low. Cells were placed in contact during 5 min. Pipette 2 was then displaced to pull the cells apart. Adhesion of the cells was detected when the doublet remained cohesive in pipette 1 after separation of the pipettes. In that case, pipette 2 was placed back into the contact of the doublet, aspiration in pipette 2 was incremented by 10 Pascal (Pa) and the pipettes were moved apart again. This cycle was repeated until the level of aspiration in pipette 2 was sufficient to separate the two cells. The separation force (SF) was obtained from the aspiration P_n−1_ and P_n_ of the two last cycles (P_n−1_ not sufficient to separate the cells and P_n_ sufficient to separate them) with equation SF = π*(d/2)^2^*(P_n−1_+P_n_)/2 (d = internal diameter of pipette 2). “Non adherent” doublets may have SF below the resolution of the technique. SF are expressed in nanoNewton (nN). Results shown were obtained from at least 4 independent experiments. The proportion of adherent doublets was compared between conditions using a Fisher's exact test.

## Results

To determine whether ORs may provide cells with adhesion properties, we chose the dual pipette assay, in which two transfected cells held by micropipettes are challenged for adhesion by putting them in contact for several minutes and thereafter trying to pull them apart (Fig. 1). The adhesion test determined: (1) whether two cells were adherent; and (2) the force necessary to separate them (separation force, SF). We used murine sarcoma S180 cells, which have already been validated for such an adhesion test [21], and indeed display very little inherent adhesion capacity in the experimental conditions we used here.

### Overexpression of MOR256-17 in S180 cells provides them with adhesion properties

**Figure 2:**
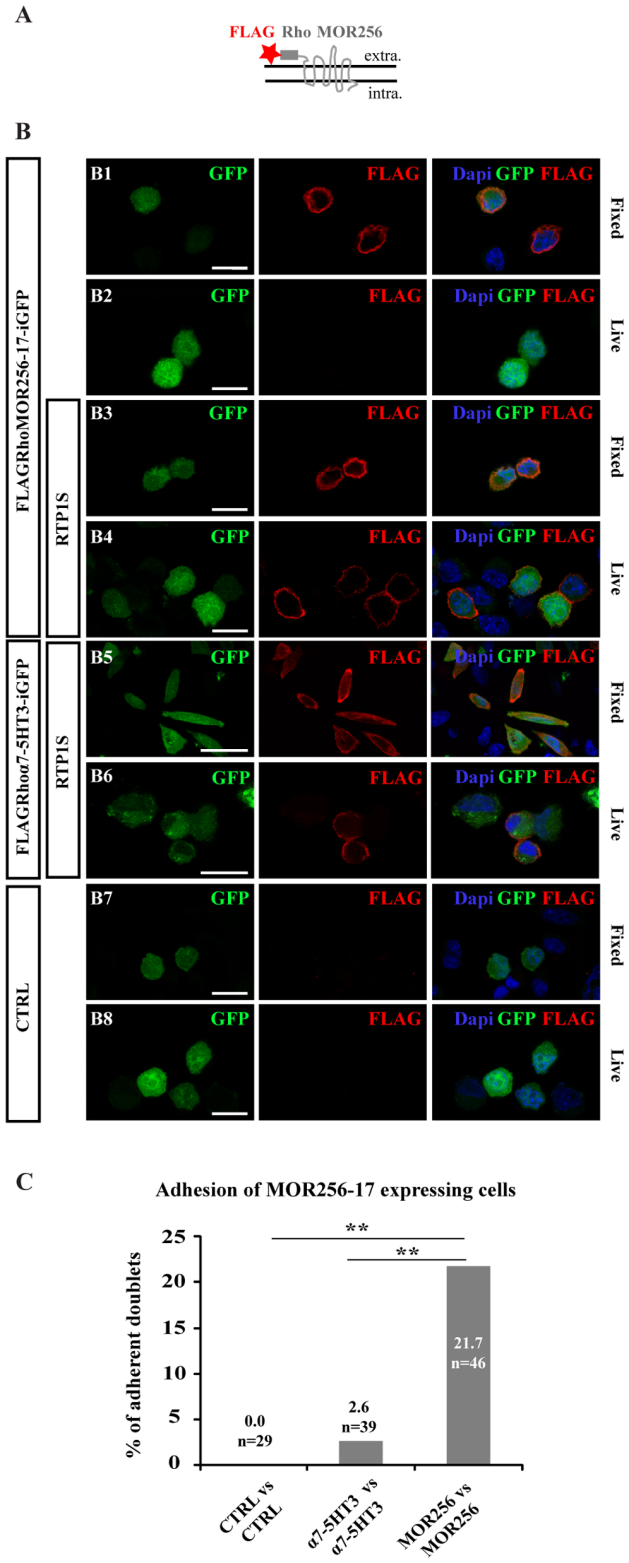
MOR256-17 overexpression in S180 cells induces cell adhesion. (A) Scheme of the FLAGRho-tagged OR shows the attachment of the FLAG tag and the Rho sequence at the extracellular (extra.) N-terminus of MOR256-17. (B) Combination of endogenous GFP fluorescence and anti-FLAG-immunostaining under fixed or live conditions, for cells transfected with pCAGGS-FLAGRhoMOR256-17-iresGFP ± RTP1S (B1–B4), pCAGGS-FLAGRhoα7-5HT3-iresGFP+RTP1S (B5–B6) or pCAGGS-iresGFP (CTRL) (B7–B8). Under live conditions, plasma membrane labeling for FLAGRhoMOR256-17 is visible only when cells are co-transfected with RTP1S (B4). Scale bar = 20 µm. (C) Percentage of adherent doublets of MOR256-17-expressing cells compared to CTRL or α7-5HT3-expressing cells. Significant number of adherent doublets is measured for MOR256-17 vs. MOR256-17 doublets, when compared to CTRL vs. CTRL or α7-5HT3 vs. α7-5HT3 doublets. ** p<0.01.

MOR256-17, one of the rare ORs previously expressed functionally in heterologous cells [22], was chosen to test in a first step if we could successfully express an OR in S180 cells. We fused MOR256-17 at its extracellular N terminus with the 20 first amino acids of the bovine rhodopsin (Rho), which improves the plasma membrane localization of ORs [23], and to a FLAG tag (Fig. 2A). The construct was cloned upstream of an iresGFP sequence in the pCAGGS plasmid, resulting in a plasmid hence named pCAGGS-FLAGRhoMOR256-17-iresGFP. We used two different constructs as negative controls: 1) a plasmid devoid of the OR sequence, the pCAGGS-iresGFP, called thereafter CTRL; and 2) the chimeric nicotinic-serotonergic α7-5HT3 receptor [18] inserted to produce a pCAGGS-FLAGRhoα7-5HT3-iresGFP plasmid. α7-5HT3 is a multi-spanning transmembrane protein belonging to the superfamily of ligand-gated ion channels. This chimeric receptor was preferred to bona fide subunits of receptors of this superfamily because it is properly addressed to the plasma membrane of heterologous cells when expressed alone, with no need for co-expression with other subunits [18]. The chimeric α7-5HT3 receptor is a non-OR transmembrane protein with no known adhesion properties. It allows us to control for potential changes in membrane protein synthesis and trafficking induced by the over-expression of exogenous transmembrane proteins and thereby assess the specificity of our observations.

Immunofluorescence analyses of fixed and permeabilized S180 cells transfected with pCAGGS-FLAGRhoMOR256-17-iresGFP showed cells displaying intracellular staining for FLAG and GFP (Fig. 2B1). However, no FLAG immunoreactivity was seen on S180 cells transfected with pCAGGS-FLAGRhoMOR256-17-iresGFP alone and processed for live and unpermeabilized immunostaining (Fig. 2B2), suggesting that no, or low levels, of MOR256-17 was addressed to the plasma membrane in these cells. We thus tested the effect of co-transfecting RTP1S (Receptor Transporting Protein 1 Short) (Fig. 2B3–B4), a protein previously reported to improve the plasma membrane localization of transfected ORs in other cell types [19], [24], [25], and in particular that of MOR256-17 in HEK293 cells [22]. Importantly, MOR256-17 appeared efficiently addressed to the plasma membrane of a subset of S180 cells when co-transfected with RTP1S, as demonstrated by the FLAG staining in live staining condition (Fig. 2B4). In parallel, the α7-5HT3 protein was properly addressed at the surface of S180 cells, as demonstrated by the presence of a subset of GFP^+^ cells displaying a clear FLAG staining at the plasma membrane (Fig. 2B5–B6). This anti-FLAG labeling was specific, since none of the CTRL-transfected cells displayed FLAG immunofluorescence, in any experimental condition (Fig. 2B7–B8).

In the dual pipette assay, while almost no GFP^+^ doublets displayed adhesion when transfected with the CTRL plasmid (0%, n = 24) or the FLAGRhoα7-5HT3-iresGFP+RTP1S constructs (one doublet out of 39; SF = 0.57 nN), 21.7% of GFP^+^ cell doublets displayed adhesion when co-transfected with FLAGRhoMOR256-17 and RTP1S (n = 46) (Fig. 2C), with a mean separation force of 1.6±0.3 nN. The difference between the adhesion ratios of the MOR256-17 group and the two control groups was statistically significant (p<0.01). Importantly, the fact that cells transfected with FLAGRhoα7-5HT3-iresGFP+RTP1S constructs displayed no significant adhesion provided strong evidence that neither RTP1S nor the potential modification of endogenous plasma membrane proteins due to ectopic protein overexpression are responsible of the measured adhesion.

Overall, these sets of data showed that the MOR256-17 OR can be expressed at the plasma membrane of S180 cells when co-transfected with RTP1S, and that introducing this OR provides these cells with significant adhesion properties measurable with the dual pipette assay.

### Another OR, M71, and the β2AR also provide adhesion properties to S180 cells

To determine if the adhesiveness induced in S180 cells by overexpressing MOR256-17 may also be provided by other ORs, we next tested M71, an OR belonging to another OR subfamily and sharing 43% amino-acid sequence identity with MOR256-17. In addition, we tested the β2AR, which is a non-OR GPCR that interestingly can substitute for an OR in mediating OSN axon fasciculation, sorting and convergence *in vivo* [8], [16].

**Figure 3:**
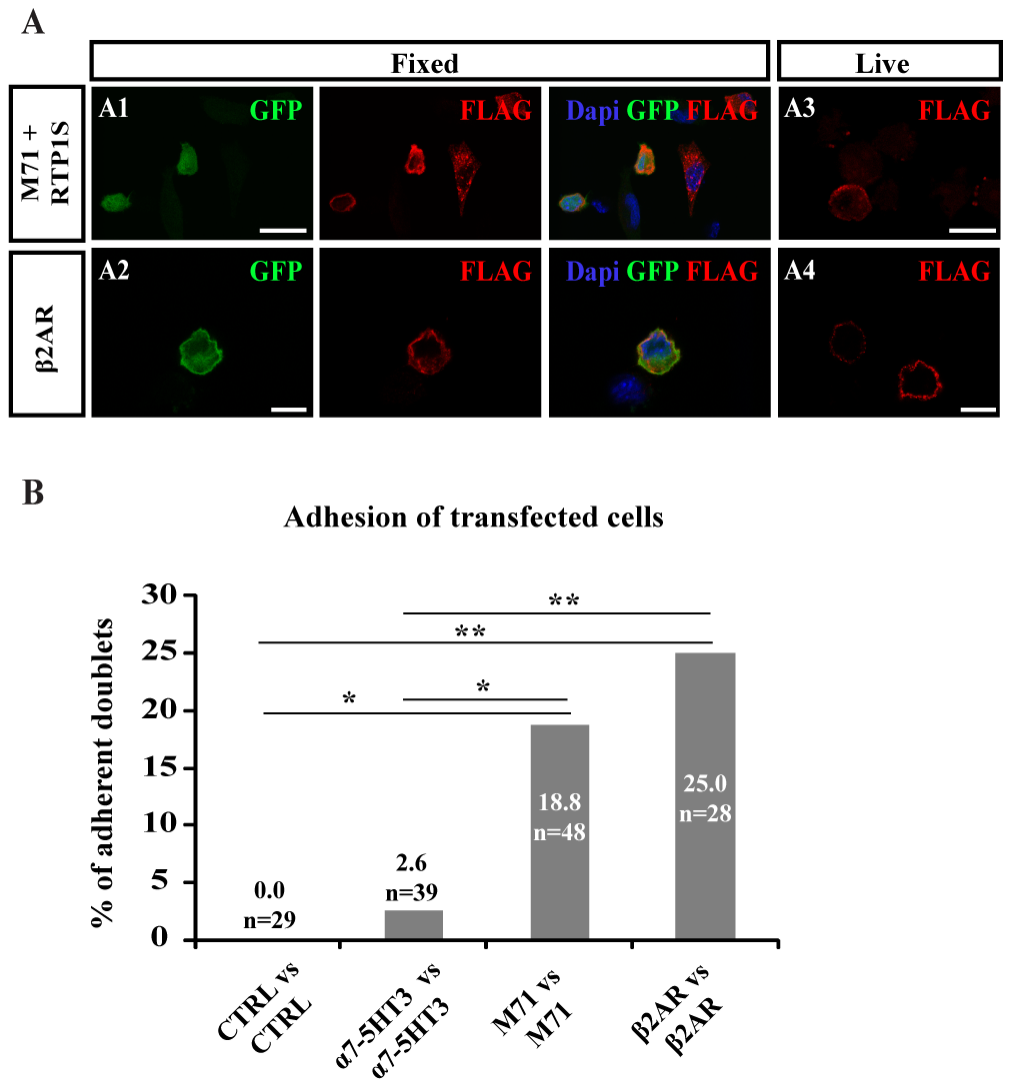
M71 or β2AR overexpression in S180 cells induces cell adhesion. (A) Combination of endogenous GFP fluorescence and anti-FLAG-immunostaining under fixed or live conditions, for cells transfected with FLAGRhoM71-iresGFP+RTP1S (A1, A3) or FLAGRhoβ2AR-iresGFP (A2, A4). Under live conditions, plasma membrane labeling for FLAG is visible in transfected cells. Scale bar = 20 µm. (B) Percentage of adherent doublets of β2AR or M71-expressing cells compared to CTRL or α7-5HT3-expressing cells. Significant number of adherent doublets is measured for β2AR vs. β2AR doublets and for M71 vs. M71 doublets, when compared to CTRL vs. CTRL or α7-5HT3 vs. α7-5HT3 doublets. * p<0.05, ** p<0.01.

Immunofluorescence analyses of fixed and permeabilized S180 cells transfected with either of the two constructs showed cells displaying co-expression of the transfected receptor and GFP (Fig. 3A1–A2). The β2AR protein was properly addressed at the surface of S180 cells, as demonstrated by the presence of a subset of GFP^+^ cells displaying a clear FLAG staining at the plasma membrane (Fig. 3A4). Consistent with MOR256-17, membrane targeting of M71 utilized the co-transfection with RTP1S (Fig. 3A3) [19], [20], [26], [27].

In the dual pipette assay, 18.8% of GFP^+^ cell doublets transfected with the FLAGRhoM71-iresGFP+RTP1S constructs displayed adhesion (n = 48; SF = 1.2±0.2 nN), and 25% of GFP^+^ cell doublets transfected with the FLAGRhoβ2AR-iresGFP construct displayed adhesion (n = 28; SF = 1.8±0.5 nN) (Fig. 3B). These data demonstrate that both M71 and the β2AR provide significant adhesion properties to S180 cells (p<0.05 for M71 and p<0.01 β2AR when compared to CTRL or α7-5HT3 doublets).

### Adhesion requires that both cells of a doublet express the GPCR, and FACS purified cells expressing the β2AR at the plasma membrane display increased frequency of adhesion

In a first step towards understanding the possible mechanisms at work in the OR- or β2AR-dependent adhesion observed in the above experiments, we first asked if a cell overexpressing the OR or the β2AR would be able to adhere to another cell which would not overexpress any of these GPCRs. For this purpose, we challenged for adhesion cells transfected with either the FLAGRhoMOR256-17-iresGFP+RTP1S or the FLAGRhoβ2AR-iresGFP constructs with cells transfected with the pCAGGS-iresGFP CTRL plasmid. In both cases, there was no significant adhesion (respectively 2.6%, n = 39, SF = 0.94 nN and 3.0%, n = 33, SF = 1.89 nN), clearly demonstrating that the expression of the GPCR is necessary in both cells of the doublet for adhesion to occur.

Since we obtained no significant adhesion with ORs which are not properly trafficked to the plasma membrane (data not shown), we hypothesized that it is necessary, for such an adhesion to occur, that the receptor successfully trafficks to the plasma membrane. To test this hypothesis, we performed our adhesion test on cell subpopulations enriched in cells that express the receptor at their surface. To do so, we purified cell populations using fluorescence-activated cell sorting (FACS) and an anti-FLAG antibody.

**Figure 4:**
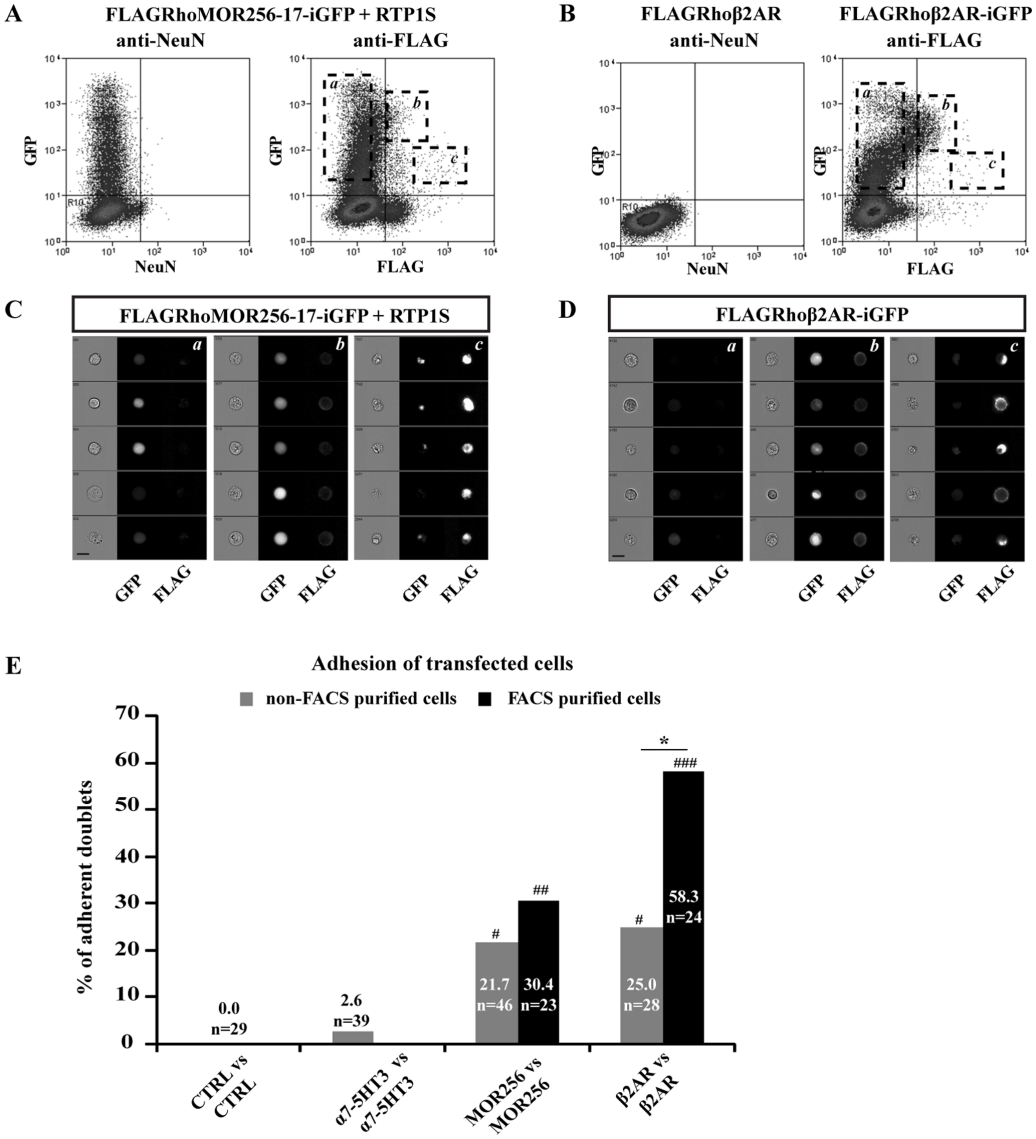
FACS purification of transfected cells increases the ratio of adherent cells. (A–B) FACS profiles of FLAGRhoMOR256-17-iresGFP+RTP1S transfected cells (A) and FLAGRhoβ2AR or FLAGRhoβ2AR-iresGFP transfected cells (B). Non-specific fluorescence was gated using isotypic control antibody (anti-NeuN) on FLAGRhoMOR256-17-iresGFP+RTP1S transfected cells and on FLAGRhoβ2AR transfected cells. FACS analysis revealed a major population of GFP^+^ FLAG^−^ cells (subpopulation *a*), as well as a smaller population of FLAG^+^ GFP^+^ double stained cells (subpopulations *b* and *c*). (C–D) ImageStream analysis of transfected cell subpopulations delineated as *a*, *b* and *c* in A and B. The subpopulations *b* were selected for purification and subsequent adhesion test, as these cells displayed a healthy morphology, cytoplasmic GFP staining and surface staining for FLAG. Subpopulations *a* and *c* were excluded from our analyses since these cells displayed no surface FLAG staining (*a*) or were dying cells (*c*). Scale bar = 15 µm. (E) Adhesion of FACS purified MOR256-17 or β2AR-expressing cells compared to CTRL or α7-5HT3 expressing cells. FACS purified cell doublets display significantly higher adhesion ratio when compared to CTRL or α7-5HT3 doublets. FACS purification significantly increases the adhesion ratio of β2AR-expressing cell doublets. * p<0.05. # p<0.05, ## p<0.01, ### p<0.001 compared to CTRL and to α7-5HT3.

Cells transfected with the MOR256-17 or β2AR constructs processed through the FACS purification protocol distributed into different categories, as shown in Fig. 4A–D. Purified cells displaying the FLAG-tagged protein at their membrane surface were allowed to recover for 18 h before performing the dual micropipette assay. Interestingly, the percentage of adherent doublets was increased to 30.4% in the population of FACS purified FLAGRhoMOR256-17+RTP1S transfected cells (n = 23; SF = 2.7±0.5 nN) and 58.3% (n = 24; SF = 2.0±0.4 nN) in the population of FACS purified FLAGRhoβ2AR transfected cells. The difference between FACS purified and non-FACS purified cell adhesion ratio (Fig. 4E) reached statistical significance for the β2AR construct, strongly suggesting that the β2AR-dependent adhesion correlates to the expression of β2AR at the cell surface. A similar trend, although not significant, was observed for the MOR256-17 construct, suggesting that purifying the cell population based on OR surface expression may slightly increase the adhesion frequency.

### Heterotypic adhesion between S180 cell doublets expressing two different ORs or one OR and the β2AR

**Figure 5:**
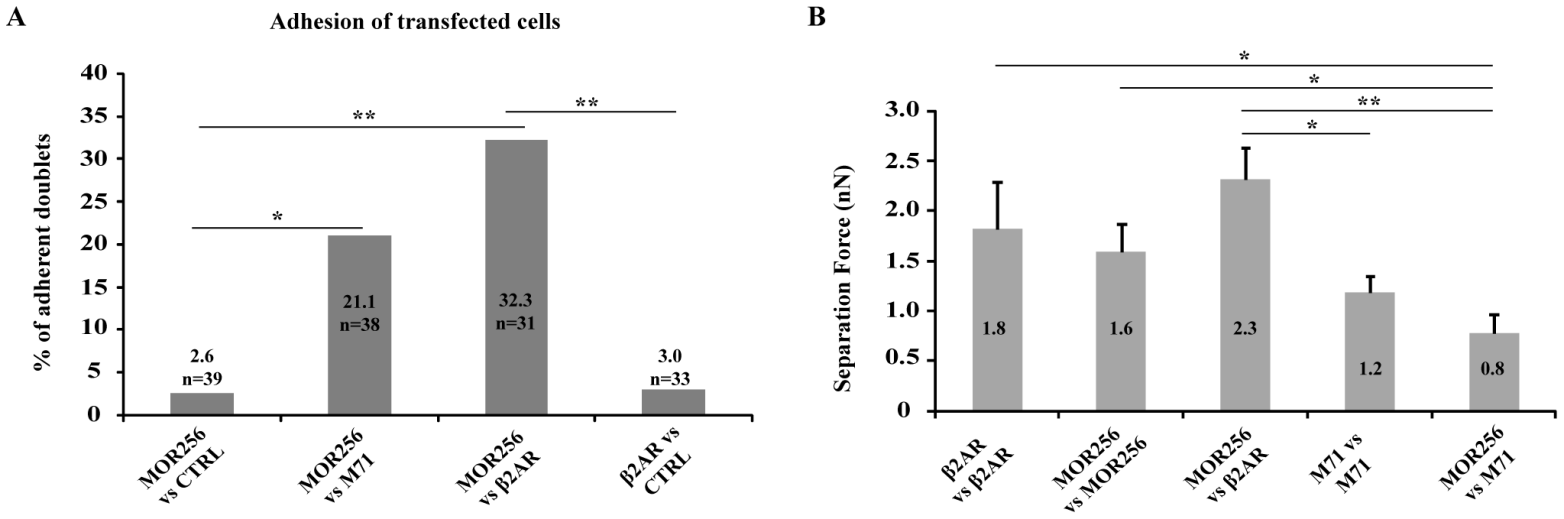
Heterotypic cell adhesion. (A) Adhesion between cells expressing two different ORs (MOR256-17 vs. M71) or an OR and β2AR (MOR256-17 vs. β2AR) compared to control heterotypic doublets (MOR256-17 vs. CTRL and β2AR vs. CTRL). A significant adhesion ratio is measured for MOR256-17 vs. β2AR doublets and for MOR256-17 vs. M71 doublets. * p<0.05. (B) Mean separation forces of homotypic and heterotypic cell doublets, Results are expressed as mean ± s.e.m. * p<0.05, ** p<0.01.

In the last series of experiments, we investigated whether heterotypic adhesion could be observed between two cells overexpressing a different receptor. Therefore, we challenged for adhesion GFP^+^ cells from MOR256-17 transfections with GFP^+^ cells from M71 or β2AR transfections. Interestingly, in both cases, a significant number of such GFP^+^ cell doublets displayed adhesion (Fig. 5A): 21.1% for MOR256-17/M71 doublets (n = 38, p<0.05 when compared to MOR256-17/CTRL doublets), and 32.3% for MOR256-17/β2AR doublets (n = 31, p<0.01 when compared to MOR256-17/CTRL and β2AR/CTRL doublets). These heterotypic adherent doublets displayed separation forces of respectively 0.8±0.2 nN for MOR256-17/M71 doublets, and 2.3±0.3 nN for MOR256.17/β2AR doublets. This set of data demonstrates that significant heterotypic cell adhesion can be induced by the overexpression of an OR in one cell, and of another OR or the β2AR in another cell.

The separation forces measured for homotypic and heterotypic doublets are in the same range of values (1.2<SF<1.8 nN for homotypic adhesion, 0.8<SF<2.7 nN for heterotypic adhesion). Statistical comparisons revealed some significant differences between the mean separation forces measured for the different groups (Fig. 5B). However, as the adhesion strength depends on the number of molecules exposed at the plasma membrane and our model does not control for expression level of the different constructs in transfected cells, comparison of the separation forces does not lead to any strong conclusions regarding the possible differences in adhesion strength under homotypic and heterotypic interactions.

In summary, our data provide evidence for OR-dependent homotypic and heterotypic adhesion, that is likely to be relevant to the wiring of olfactory sensory axons projections.

## Discussion

We report here that overexpression of ORs or β2AR induces adhesion of both homotypic and heterotypic cell doublets, with separation forces in the nanoNewton (nN) range. No such adhesion was induced by overexpressing in these cells another plasma membrane protein composed of several transmembrane domains but belonging to another family of receptors, the α7-5HT3 protein, demonstrating the specificity of the effects observed with the GPCRs we tested here. This is the first direct evidence that adhesion, mediated by OR expression, may contribute to wiring in the olfactory system. Given the current technical challenges of investigating the OR-dependent adhesion directly on OSN axons *in vivo*, we designed a new *in vitro* approach in which ORs are overexpressed in a cell line suitable for adhesion testing using a biophysical assay.

Testing the adhesion capacity of ORs required the demonstration of the proper trafficking of ORs to the plasma membrane of heterologous cells. Although functional expression of ORs has been a recurrent issue in the field [26], [27], we circumvented this problem in S180 cells by co-transfecting a FLAG-tagged and Rho-fused MOR256-17 or M71 construct with RTP1S, combining previously published strategies [8], [19], [22], [23], [27]. We were then able to obtain the surface expression of MOR256-17 and M71 in a subset of transfected cells.

From a mechanistic point of view, the homotypic and heterotypic adhesion of cells expressing ORs or β2AR we report here might be due to: 1) the regulated expression of *bona fide* adhesion molecules through the activation of the GPCRs and its cAMP signaling pathway [10], [11]}; or 2) the direct trans-interactions of GPCRs themselves or indirect trans-interactions of GPCR-containing multimolecular complexes, as proposed by Feinstein and Mombaerts (2004). Since it is very unlikely that odorants or agonists for the ORs tested here (as well as agonists for the β2AR) are present in the culture medium, we exclude the possibility that the observed adhesion involves conventional ligand-induced activation of the GPCRs, followed by the expression of *bona fide* adhesion molecules. However, we cannot rule out that constitutive activity of the overexpressed GPCR in the transfected cells may be sufficient to trigger the cAMP pathway. Interestingly, constitutive activity of ORs was recently proposed to play a role in OSN axon targeting along the anterior-posterior axis of the olfactory bulb [28]. Nevertheless, it appeared to have no significant effect on the expression of molecules known to play a critical role in glomerular segregation, such as the kirrel molecules [28]. Alternatively, it has been suggested that OR-dependent homotypic interactions between axons may modulate the cAMP cascade activity, thus providing a link between interacting ORs and intracellular pathways [8]. Although a modulation of cAMP may occur upon cell contact in our GPCR overexpressing cells, it seems unlikely that the 5 min apposition time would be sufficient for a cAMP modulation to activate the transcription and functional expression of adhesion molecules. The low separation forces characterizing our GPCR-dependent adherent cell doublets (0.8±0.2 to 2.3±0.3 nN depending on the GPCR tested) suggest that the adhesion may not involve conventional adhesion molecules displaying high adhesion properties such as E-Cadherin, which in similar experimental conditions displayed separation forces of higher magnitude (i.e. >50 nN [21]), but they are close to the range of forces characterized by other adhesion molecules like Cad11 or CX3CR1-fractaline (6-7.4 nN) [29], [30] However, since the separation force measured in our assay strongly depends on the number of adhesion molecules expressed by the cells, the low separation forces characterizing our adherent doublets may also be the result of an OR-dependent expression of conventional adhesion molecules at very low levels.

Several of our results also support the hypothesis of trans-interaction between GPCRs or GPCR-containing complexes. Indeed, the GPCR has to be expressed, and most probably present at the surface of both cells, to allow adhesion. Doublets composed of a GPCR overexpressing cell and a CTRL-transfected cell did not adhere, and FACS purification of the cells expressing the transfected GPCR at their surface leads to an increased adhesion ratio. It will be critical to determine in future experiments if ORs trans-interact directly and if so, how these interactions occur. Indeed, ORs (as well as the β2AR) are classical GPCRs, devoid of those large N-terminal regions that contain classical adhesion domains characterizing recently identified GPCR members of the “adhesion-GPCRs” subfamily [31].

From a functional point of view, what could be the role of OR-induced adhesion in the wiring of olfactory sensory projections? As OSN axons exit the olfactory epithelium, they initially fasciculate with nearest neighbors, not necessarily with axons from other OSNs expressing the same OR [32]. However, as they progress into the OB nerve layer, these axons undergo a profound topographical reorganization so that all the axons emerging from OSNs expressing the same OR fasciculate homotypically and converge into only 2 or 3 glomeruli per OB [5], [33]. This multistep developmental process most likely involves several molecular mechanisms including previously identified OR-independent and -dependent pathways (e.g. [34] for review). We propose that the OR-dependent adhesion uncovered in the present work is an additional mechanism by which axonal ORs, which may be locally synthesized in axons [35], may favor the formation of - or stabilize - fascicles of axons expressing the same OR, hence facilitating their sorting. In our model, the final sorting of OSN axons would be the result of dynamic axon-axon interactions in which both homo- and heterotypic interactions may mediate adhesion [36]. Given the homotypic fasciculation of OSN axon subpopulations *in vivo*, we had hypothesized that only homotypic cell doublets tested in our assay would display adhesiveness. The adhesion between heterotypic cells was unexpected because axons expressing different ORs need to segregate from each other, a process that could have been ensured by non-adhesion or repulsion. Further experiments with other ORs will be necessary to determine if heterotypic adhesion is a general feature, but it may well be that differential adhesion - rather than strictly homotypic adhesion - may be used to ensure the OR-dependent sorting of the olfactory axon populations. The highest degrees of adhesion would be provided by homotypic interactions, thus stabilizing bundles of axons displaying the same identity, at the expense of heterotypic fascicles. In S180 cells, we did not observe significantly lower separation forces for heterotypic doublets, compared to homotypic ones. However the experiment we designed may not fully recapitulate the fine regulations that may occur *in vivo* in OSN axons, as transiently transfected S180 cells express GPCRs at levels that vary considerably from one cell to another and from one GPCR to another, thereby influencing separation forces. Nevertheless, such a concept of differential adhesions, with adhesion forces correlated to the degree of OR similarity, is fully in line with previous observations that OSN expressing homologous OR genes within a cluster tend to project their axons to very close but distinct subsets of glomeruli [37], and that axons expressing a point-mutated OR often coalesce into a glomerulus located close to the wild-type OR glomerulus [14], [16].

Finally, we show here that two ORs (MOR256-17 and M71) and the β2AR can mediate homotypic adhesion, raising the question whether this property may be generalized to the GPCR family. Interestingly, another GPCR, the type 1 Cannabinoid Receptor is expressed along developing axon bundles in the brain, where it may play a role in axon guidance and fasciculation (reviewed in [38]). Given the observations made in the present study, it will be interesting to re-examine the expression of GPCRs during the development of axonal projections, and to investigate their possible functions in axonal fasciculation through adhesion properties.
